# Molecular Machines Regulating the Release Probability of Synaptic Vesicles at the Active Zone

**DOI:** 10.3389/fnsyn.2016.00005

**Published:** 2016-03-02

**Authors:** Christoph Körber, Thomas Kuner

**Affiliations:** Department of Functional Neuroanatomy, Institute of Anatomy and Cell Biology, Heidelberg UniversityHeidelberg, Germany

**Keywords:** release probability, synaptic vesicles, active zone, calyx of Held, short-term synaptic plasticity, calcium channels

## Abstract

The fusion of synaptic vesicles (SVs) with the plasma membrane of the active zone (AZ) upon arrival of an action potential (AP) at the presynaptic compartment is a tightly regulated probabilistic process crucial for information transfer. The probability of a SV to release its transmitter content in response to an AP, termed release probability (P_r_), is highly diverse both at the level of entire synapses and individual SVs at a given synapse. Differences in P_r_ exist between different types of synapses, between synapses of the same type, synapses originating from the same axon and even between different SV subpopulations within the same presynaptic terminal. The P_r_ of SVs at the AZ is set by a complex interplay of different presynaptic properties including the availability of release-ready SVs, the location of the SVs relative to the voltage-gated calcium channels (VGCCs) at the AZ, the magnitude of calcium influx upon arrival of the AP, the buffering of calcium ions as well as the identity and sensitivity of the calcium sensor. These properties are not only interconnected, but can also be regulated dynamically to match the requirements of activity patterns mediated by the synapse. Here, we review recent advances in identifying molecules and molecular machines taking part in the determination of vesicular P_r_ at the AZ.

## Introduction

Information transfer in the nervous system relies on the precisely timed release of neurotransmitter from the presynaptic compartment once an action potential (AP) arrives. The arrival of the AP causes the depolarization of the presynaptic plasma membrane which in turn leads to the opening of VGCCs, resulting in a calcium influx into the presynaptic compartment during the repolarization phase of the AP. The calcium ions entering the cell subsequently interact with a calcium sensor located at the synaptic vesicles (SVs) and trigger the fusion of one or more SVs with the presynaptic plasma membrane, thereby releasing neurotransmitter into the synaptic cleft (Lisman et al., 2007; Rizzoli, 2014). Importantly, this basic mechanism is a highly probabilistic, yet tightly regulated, process. In a typical forebrain synapse, only ~15% of APs arriving at the presynaptic terminal cause fusion of a SV (Branco and Staras, 2009; Borst, 2010). Thus, regulating P_r_ of a given synapse is a powerful mechanism to adjust information transfer to specific requirements of the circuit, as it provides the basis for information coding by synchronous activity of a population of neurons (low P_r_), as well as for the faithful transmission of information on a one-to-one basis (high P_r_). Differences in P_r_ do not only occur between different types of synapses in functionally different circuits, but also among presynaptic compartments of the same axon, leading to a situation in which the presynaptic compartments of a given neuron vary in their P_r_, depending on the postsynaptic partner and the functional state of the particular synapse (Atwood and Karunanithi, 2002). Additionally, P_r_ is not even homogeneous for SVs of the same presynaptic terminal (Neher, 2015).

Therefore, we would like to address the following questions: how is P_r_ regulated at the presynaptic compartment and what mechanisms cause differences in the P_r_ between single SVs of the same presynaptic termial? We review recent advances in identifying the molecular machines involved in the various mechanisms controlling the P_r_ of a presynaptic SV at central mammalian synapses. This includes the availability of release-ready SVs, the process of SV fusion, calcium influx and calcium handling. Moreover, we will discuss changes of these parameters that may occur during ongoing synaptic activity.

## The Readily Releasable Pool

The basis for every form of synaptic neurotransmitter release is the availability of SVs that are competent for immediate fusion on the time scale of microseconds, once an AP arrives at the presynapse. However, only a small fraction of the SVs present at a presynaptic compartment possess fusion competence. SVs associated with an active zone (AZ) can be divided, according to the classic model, into three different functional pools: the readily releasable pool (RRP), the recycling pool and the reserve pool (Rizzoli and Betz, 2005; Denker and Rizzoli, 2010). Here, we focus on the SVs of the RRP that are thought to be in close contact with the plasma membrane at the site of SV fusion and ready to immediately release transmitter upon arrival of an AP (Rizzoli and Betz, 2005; Denker and Rizzoli, 2010). However, the RRP is not a uniform population of SVs and depending on the synapse, can be further subdivided into a fast and a slow releasing subpopulation (Wu and Borst, 1999; Sakaba and Neher, 2001; Neher, 2015). Furthermore, it is controversial whether all three forms of synaptic release (synchronous, asynchronous and spontaneous release, for a recent review, see Kaeser and Regehr, 2014) originate from the same pool of readily releasable SVs, or whether there is a distinct population of SVs that exclusively supplies spontaneous release (Kavalali, 2015; Schneggenburger and Rosenmund, 2015, respectively). Here, we will focus on mechanisms that maintain the RRP available for AP-induced release. The size of the RRP, defined as the number of SVs in close contact with the plasma membrane and immediately available for release, is determined by various molecular machines of the presynaptic compartment. SVs that are part of the RRP need to be transported to the AZ, anchored there and subsequently rendered fusion competent. The latter two steps are known as docking and priming, respectively. Proteins interfering with either of these steps will alter the number of release-ready SVs and thereby the P_r_ of individual SVs. Non-docked and primed SVs need to undergo molecular steps in order to become fusion-competent and thereby join the RRP. Moreover, the P_r_ of the entire synapse will be changed, because the size of the RRP, and thus the reservoir of SVs that can potentially be released, is altered.

## Recruitment of SVs to the RRP

The first step in the mechanism of SV recruitment to the RRP is the delivery of SVs to the site of release, a process that has been studied intensively at the calyx of Held synapse in the auditory brainstem. There, the polymerization of actin has been identified as one of the main factors for the recruitment of SVs to the RRP, especially to the so-called fast-releasing subpool that consists of the SVs immediately available to fusion upon AP-arrival (Sakaba and Neher, 2003b; Lee et al., 2012, 2013). In addition to actin, myosin light chain kinase (MLCK), another cytoskeleton-associated protein, was found to regulate recruitment of SVs to the RRP. At the calyx of Held, inhibition of MLCK resulted in an increase in RRP size with a specific augmentation of its fast-releasing subpool (Srinivasan et al., 2008). On the other hand, activation of MLCK and thus phosphorylation of MLC triggers the contraction of actomyosin in the presynaptic compartment (Luo, 2002) and reduces the number of SVs in the RRP in hypoglossal motorneurons (García-Morales et al., 2015). MLCK in turn is negatively regulated by Rho-associated kinase (ROCK) and basal ROCK activity is necessary to maintain the RRP (González-Forero et al., 2012). A third link between the recruitment of SVs to the RRP and the actin cytoskeleton comes from a recent study demonstrating that the endocytic scaffolding protein intersectin 1 and its interaction with Cdc42, a GTPase involved in actin remodeling, are necessary for the replenishment of SVs to the RRP (Sakaba et al., 2013). Thus, the actin cytoskeleton and its regulators have a profound effect on the recruitment of SVs to the RRP.

Beside this cytoskeletal machinery, two enzymes play opposing roles in the regulation of the RRP size: cyclin-dependent kinase 5 (CDK5) and the calcium activated phosphatase calcineurin (CaN). Whereas CDK5 activity limits the number of SVs in the RRP, activation of CaN, possibly in response to ongoing synaptic activity, recruits SVs to the RRP and increases its size (Kim and Ryan, 2010; Marra et al., 2012). Relatively little is known about the target proteins that are modified in their phosphorylation state by these two enzymes. CDK5 has been shown to phosphorylate synapsin I which leads to increased SV clustering distant to the AZ, and thus outside the RRP. This clustering is supposed to be mediated by an increased binding of phosphorylated synapsin I to F-actin (Verstegen et al., 2014). Specific targets for CaN in regulating RRP size have not been reported till date, as CaN is mostly known for triggering bulk endocytosis by dephosphorylation of dynamin (Clayton et al., 2009). However, both, CDK5 and CaN have been proposed to regulate the phosphorylation of N-type VGCCs (Ca_v_2.2; Su et al., 2012; Kim and Ryan, 2013). Pharmacological blockade or knock-down of CDK5 resulted in an increase in calcium influx through N-type VGCCs and a concomitant increase in SV exocytosis, whereas block or knock-down of CaN had the opposite effect (Kim and Ryan, 2013). Contradictory to this mechanism, it has been reported, using CDK5 phosphorylation-insensitive N-type channel mutants, that phosphorylation of the N-type VGCC increases calcium influx and number of SVs at the AZ, possibly via a direct interaction with the presynaptic scaffolding protein RIM1 (Rab 3 interacting molecule 1; Su et al., 2012). Under physiological conditions the regulation of the balance between CDK5 and CaN is currently not known, although CaN activation and a subsequent increase in RRP size have been reported to depend on postsynaptic N-Methyl-D-aspartate (NMDA) receptor activation and retrograde signaling via NO (Ratnayaka et al., 2012). Moreover, the size of the RRP is determined directly by the influx of calcium through VGCCs during synaptic activity, as higher calcium influx and a larger local calcium domain lead to a rise in calcium concentration sufficient for SV fusion at larger distances from the VGCC (Thanawala and Regehr, 2013).

Finally, size and replenishment of the RRP are dynamically regulated by the level of synaptic activity. In the auditory brainstem, unilateral hearing loss results in a reduction in RRP size at the calyx of Held (Grande et al., 2014), while constant noise exposure induced a reversible increase in RRP size at the endbulb of Held (Ngodup et al., 2015). Of note, an earlier study on Ca_v_1.3-KO mice, which are deaf due to non-functional glutamate release from inner hair cells, did not reveal changes in RRP size (Erazo-Fischer et al., 2007).

## Docking and Priming of SVs at the AZ

Once SVs have been recruited to the AZ, they need to be anchored and rendered fusion competent. This process depends on several different molecular machines. The most obvious one is the SNARE complex, as its formation is a prerequisite for all forms of membrane fusion (see separate section below, reviewed by Jahn and Fasshauer, 2012; Südhof, 2013). Additionally, scaffolding proteins of the cytomatrix of the AZ and proteins present on the SVs take part in this process (Takamori et al., 2006; Südhof, 2012).

The scaffolding proteins of the AZ are thought to ensure the correct structural assembly of the release site, including the docking of SVs to the plasma membrane of the AZ. Recent studies using electron tomography have shown that SVs in the vicinity of AZ are connected to the plasma membrane via short filamentous structures called tethers (Siksou et al., 2007; Fernández-Busnadiego et al., 2010). The number of these short tethers as well as the quantity and distribution of SVs close to the AZ are altered in RIM1α-knock-out synapses, suggesting a role for RIM1α in adhering/docking SVs to the AZ (Fernández-Busnadiego et al., 2013). Functional evidence that RIM proteins are crucial for RRP maintenance comes from different preparations. At the calyx of Held and in hippocampal, bouton-type synapses, simultaneous knock-out of RIM1 and RIM2 leads to an almost complete loss of SVs close to the AZ. This in turn caused a severe impairment of synaptic transmission (Han et al., 2011; Kaeser et al., 2011). However, both RIM isoforms seem to be redundant, at least to a certain degree (Kaeser et al., 2012; Han et al., 2015). The mechanism by which RIM assists in the docking of SVs to the AZ and thereby recruits them to the RRP involves the interaction with another AZ protein: Munc13. In the absence of RIM, Munc13 forms an autoinhibitory homodimer that is relieved by the interaction with RIM (Deng et al., 2011). RIM, Munc13 and the SV protein Rab3 can then form a tripartite complex (Dulubova et al., 2005) that links SVs to the cytomatrix of the AZ and thereby positions the SV in close proximity to the presynaptic plasma membrane. Munc13 is absolutely crucial for SV fusion, as the simultaneous genetic removal of the isoforms Munc13-1 and Munc13-2 prevents every form of neurotransmitter release (Varoqueaux et al., 2002). Synapses devoid of Munc13-1/2 are characterized by the loss of SVs docked to the AZ and thus of the RRP (Siksou et al., 2009; Imig et al., 2014). However, SVs tended to accumulate close to the AZ (~10 nm) in the absence of Munc13-1/2, indicating that SVs are recruited to the AZ but do not docked to the plasma membrane (Imig et al., 2014). Moreover, synapses express multiple Munc13 isoforms simultaneously that may execute different functions in the recruitment of SVs to the RRP. At the calyx of Held, overexpression of a dominant negative form of Munc13-1, the major isoform at this synapse, results in a severe reduction in RRP size, whereas simultaneous deletion of Munc13-2 and Munc13-3 had no effect on RRP size, but slowed down the replenishment of the RRP after intense activity (Chen et al., 2013). Munc13 has also been shown to prime SVs and thereby render them fusion competent. This involves the interaction of Munc13 with the calcium binding protein calmodulin (CaM; Junge et al., 2004). Rendering the Munc13-1 binding site for CaM nonfunctional impairs the recruitment of SVs to the AZ and their incorporation into the RRP (Lipstein et al., 2013). In addition to the CaM-dependent replenishment of docked and primed SVs in the RRP, Munc13 has also been proposed to be necessary for a molecular process of unknown nature called “superpriming”. This reaction converts primed SVs of the RRP into SVs ready for immediate fusion within microseconds after the arrival of the AP, independent of CaM or actin (Lee et al., 2013; Ishiyama et al., 2014).

In addition to RIM and Munc13, other AZ proteins such as CAPS1 and CAPS2 have been implicated in docking and priming SVs of the RRP. The combined loss of both, CAPS1 and CAPS2, results in a strong reduction in SVs present at the AZ (Jockusch et al., 2007; Imig et al., 2014), but unlike Munc13-1/2 knock-out synapses, CAPS1/2 knock-out synapses do not accumulate SVs close to the AZ. This suggests that CAPS1/2 are not only required for docking and priming of SVs, essential steps in the generation of the RRP, but also for the recruitment of SVs to the AZ (Imig et al., 2014). Furthermore, the presynaptic scaffolding protein liprin-α2 has been shown to regulate the RRP size. Knock-down of liprin-α2 in cultured hippocampal neurons led to a severe reduction in the number of docked SVs at the AZ and structural remodeling of the AZ, including shortening and reduction of the amount of RIM present. Therefore, the diminished number of docked SVs upon the loss of liprin-α2 could be secondary to the reduction in RIM at the AZ (Spangler et al., 2013). Interestingly, mSyd1A (mouse Synapse-Defective-1A) that interacts with liprin-α2 and with Munc18 (see below) has also been shown to be involved in SV docking (Wentzel et al., 2013).

In addition to these AZ components, other proteins present on SVs have been implicated in the docking and priming of SVs at the AZ. An obvious candidate for the involvement in SV docking at the AZ is Rab3, as it interacts with both, the major scaffolding protein RIM and SVs (Wang et al., 1997). However, knock-out of all four Rab3 isoforms (Rab3A-D) did not result in a loss of docked SVs but in a profound reduction in P_r_. This suggests that Rab3 is involved in priming rather than docking of SVs (Schlüter et al., 2004). More specifically, Rab3 seems to play a role in superpriming since it increases the calcium sensitivity of docked and primed SVs, making it more likely that they get released in response to an AP (Schlüter et al., 2006). Rab3 may therefore be part of the same molecular machine as Munc13 (Lee et al., 2013). Another SV protein that has been implicated in the regulation of the RRP size is SV2. Genetic deletion of the SV2A isoform has been reported to reduce RRP size (Custer et al., 2006), whereas another study reported a normal RRP size and attributed the observed reduction in P_r_ to a deficit in the responsiveness of primed SVs to calcium influx (Chang and Südhof, 2009). Along similar lines, Mover, a newly described SV protein that modulates P_r_ in a subset of central synapses, may be involved in the superpriming of SVs, as its knock-down increases P_r_ at the calyx of Held, without altering RRP size (Körber et al., 2015).

## Replenishment of The RRP

Upon the arrival of an AP, fusion competent SVs of the RRP are released at the AZ. To cope with ongoing synaptic activity, the RRP needs to be constantly replenished with newly recruited, docked and primed SVs. Therefore, molecular interactions that interfere with SV replenishment will impair the maintenance of the RRP and thus decrease the P_r_ of the synapse during sustained activity. Deficits in RRP replenishment often only become evident during high frequency synaptic activity and are, accordingly, particularly prominent at synapses that participate in high frequency signaling under physiological conditions, like the calyx of Held or the cerebellar mossy fiber terminals. A variety of presynaptic components have been implicated in RRP replenishment: presynaptic scaffolding proteins, ATP and the spread of the presynaptic calcium domain upon calcium channel opening.

Bassoon, a large multi-domain presynaptic scaffolding protein has been shown to play a crucial role in the refilling of the RRP at high frequency-firing synapses such as the cerebellar mossy fiber to granule cell synapse and the endbulb of Held in the auditory brainstem (Hallermann et al., 2010; Mendoza Schulz et al., 2014, respectively). Interestingly, in synapses with lower physiological activity, like those connecting hippocampal neurons, the loss of bassoon had no effect on RRP replenishment (Altrock et al., 2003; Mukherjee et al., 2010). Furthermore, loss of bassoon also lacked a functional phenotype in hippocampal neurons when the closely related protein piccolo was knocked-out simultaneously, even though the number of SVs per synaptic terminal was severely reduced (Mukherjee et al., 2010). Piccolo in turn has been proposed to modulate the dynamics of synapsin 1 (Leal-Ortiz et al., 2008). However, knock-out of all three synapsin genes at the calyx of Held resulted in a general reduction of SVs in the presynaptic compartment but not in the RRP (Vasileva et al., 2012), similar to what has been seen at hippocampal bassoon/piccolo double knock-out synapses (Mukherjee et al., 2010). Nevertheless, the lack of all three synapsins led to a slowing of RRP replenishment at the calyx of Held during high frequency signaling (Vasileva et al., 2012). Interestingly, knock-down of piccolo in hippocampal cultured neurons led to a reduced stability of F-actin and thereby to altered synapsin dynamics, linking piccolo to the regulation of SV availability at the AZ (Waites et al., 2011). Of note, a recent imaging study of dissociated calyces of Held suggested that SVs newly recruited to the AZ are not immediately used to replenish the RRP. Instead, SVs have to reside at the AZ for a certain amount of time before they are rendered fusion competent and used to replenish the RRP during ongoing activity (Midorikawa and Sakaba, 2015).

Another important regulator of RRP replenishment is calcium. It has been shown that the increase in presynaptic calcium concentration during high frequency synaptic activity speeds-up the refilling of the RRP at the calyx of Held (Wang and Kaczmarek, 1998). This effect is presumed to be mediated by binding of calcium to CaM. CaM has been shown to be involved in replenishing the fast-releasing subpool of the RRP responsible for maintaining high frequency synaptic transmission (Sakaba and Neher, 2001; Hosoi et al., 2007). This mechanism may predominantly depend on the interaction between CaM and the priming factor Munc13 (Lee et al., 2012; Lipstein et al., 2013). However, CaM also participates in various forms of SV endocytosis (Wu et al., 2009; see below) and has therefore been suggested to link SV endocytosis to replenishment of the RRP (Yao and Sakaba, 2012). Interestingly, the CaM-dependent priming of SVs can be regulated by presynaptic GABA_B_ receptors (GABA_B_Rs) at the calyx of Held. Activation of GABA_B_Rs resulted in a decrease in cAMP that counteracted the activating effect of elevated presynaptic calcium concentration on SV priming (Sakaba and Neher, 2003a). Moreover, replenishment of the RRP has been shown to be strongly dependent on locally produced ATP. ATP maintains the SV cycle and ATP deprivation leads to severe impairments in neurotransmitter release during ongoing activity (Rangaraju et al., 2014; Pathak et al., 2015). Interestingly, ATP also binds to synapsins that are involved in the regulation of RRP size. Deficiency in ATP-binding of synapsins has been reported to lead to a larger initial RRP size, but a slower replenishment (Orlando et al., 2014; Shulman et al., 2015). Besides these proteins and intracellular molecules, an additional factor that controls RRP replenishment is temperature, as raising the temperature increases the SV replenishment rate at the calyx of Held, although temperature has no effect on RRP size and the initial P_r_ of SVs (Kushmerick et al., 2006).

Thus, the establishment and maintenance of the RRP is a highly regulated process involving a large number of molecular machines, ranging from the actin cytoskeleton to the generation of ATP, that are highly interconnected and act together in order to provide the synapse with enough SVs to cope with the demands of synaptic activity. Interference with any of the different molecular machines leads to an impairment in SV recruitment to the RRP and thereby, to a decrease in synaptic P_r_.

## The SNARE Complex and associated Proteins

The SNARE complex is the molecular machine that provides the energy for SV fusion with the plasma membrane (Jahn and Fasshauer, 2012; Südhof, 2013). Besides this, the SNARE complex is also implicated in the docking and priming of SVs to the AZ, and the loss of either of the t-SNAREs syntaxin 1 and SNAP25 results in the loss of SVs that are in close contact with the presynaptic plasma membrane. The situation is a bit more difficult for the v-SNARE synaptobrevin (a.k.a. VAMP2), since the analysis of synapses devoid of synaptobrevin is hampered by the compensatory upregulation of VAMP1 which can, at least partially, take over the function of synaptobrevin (Imig et al., 2014). Nevertheless, acute disruption of synaptobrevin function by tetanus toxin blocked SV release completely at the calyx of Held (Sakaba et al., 2005). Moreover, the proline-rich domain of synaptobrevin has been shown to be involved in the recruitment of SVs into the fast-releasing subpool of the RRP, possibly by positional priming (Wadel et al., 2007). Positional priming is a mechanism that is thought to be responsible for the positioning of docked SVs in close proximity to VGCCs. This ensures a short distance between the SV and the source of calcium (coupling distance) and thereby the rapid release of SVs in response to VGCC opening (see below). Of note, syntaxin 1 has been proposed to not only play a role in the docking and priming of SVs to the RRP (Arancillo et al., 2013), but also to speed-up SV fusion when present in its open conformation (Acuna et al., 2014). However, during the process of SV fusion, the SNARE complex not only needs to be formed, but the so called four-helix bundle needs to be positioned at the right distance from the plasma membrane in order to render the SNARE complex functional (Zhou et al., 2013).

Apart from the three canonical SNARE complex proteins syntaxin 1, synaptobrevin and SNAP25, a number of other proteins have been implicated in the fusion of SVs through their interactions with the SNARE complex. One of these proteins is complexin, whose role in SV release has been discussed controversially in recent years. Complexin has been proposed to either facilitate release or act as a fusion clamp (Brose, 2008; Südhof, 2013). Apart from this highly debated role in SV fusion, complexins (isoforms 1 and 2) have a distinct role in priming SVs and thereby rendering them release-ready (Yang et al., 2013; Chang et al., 2015), a function probably exerted by its C-terminus (Kaeser-Woo et al., 2012). The functional role of complexins in SV priming has been suggested to be the stabilization of newly primed SVs, which would correspond to a fusion clamp mechanism (Chang et al., 2015). However, this function may depend on the type of synapse studied, the examination method used and the developmental history of the neuron (Yang et al., 2013). Complexins have also been implicated to be involved in determining the size of the RRP (Kaeser-Woo et al., 2012; Yang et al., 2013), although this may not apply to all synapses (Chang et al., 2015). Thus, although the exact mechanisms by which complexins act are still unclear, it appears that complexins are involved in the stabilization of the primed state of the SV thereby preventing untimely fusion and thus regulating synaptic P_r_ by maintaining the RRP.

Another protein interacting with the SNARE complex that is crucial for SV fusion is Munc18-1. The genetic deletion of Munc18-1 leads to the total arrest of all forms of neurotransmitter release although the ultrastructure of the synapses appears normal (Verhage et al., 2000). Munc18-1 binds to syntaxin1 in its closed conformation and is thought to stay bound to the assembled SNARE complex where it assists in the zippering process in a synaptobrevin-dependent manner (Shen et al., 2015). But in order to form, the SNARE complex requires the interaction of the Munc18-1/syntaxin1 complex with additional factors, e.g., Munc13 (Rizo and Rosenmund, 2008; Ma et al., 2013; Südhof, 2013; Kavanagh et al., 2014). However, Munc18-1 critically influences the P_r_ of a synapse, as the amount of Munc18-1 present at a synapse scales with its strength (Toonen et al., 2006; Cijsouw et al., 2014), presumably by determining the number of SNARE complexes that can be formed and thus the number of release-ready SVs (Toonen et al., 2006).

Munc18-1 is assisted in the formation of the SNARE complex by Munc13 (Ma et al., 2013), that, besides its role in SV docking and priming described above, has additional effects on the SNARE complex once formed. Munc13 contains a C1-domain that binds to diacylglycerol (DAG) produced by activation of phospholipase C (PLC). DAG binding has no effect on the basal docking and priming activity of Munc13 and therefore on the RRP size, but activation of the C1-domain leads to an increase in vesicular P_r_ by reducing the energy barrier for SV fusion (Basu et al., 2007). Modulation of the energy barrier for SV fusion has been recently shown to be a potent way to regulate the P_r_ of SVs in general and is not restricted to Munc13, but has also been shown for complexins (Schotten et al., 2015).

Precisely timed, synchronous release of SVs of the RRP upon arrival of an AP at the presynaptic terminal is dependent on synaptogmin (isoforms 1, 2 or 9, depending on the brain region; Xu et al., 2007), the principal calcium sensor for synchronous release. The exact mechanisms of synaptotagmin action are still under debate, although they have been extensively studied in recent years (Chapman, 2008; Südhof, 2013; Zhou et al., 2015). Synaptotagmin possesses two calcium binding C2-domains. Calcium binding to the C2B-domain has been shown to be crucial for synchronous SV fusion (Nishiki and Augustine, 2004) whereas the C2A-domain has only an accessory role in this process (Shin et al., 2009). Additionally, the C2B-domain interacts directly with the lipids of the presynaptic plasma membrane and the SNARE complex (Rickman et al., 2004; Xue et al., 2008, respectively), which provides additional support for the fusion reaction. In particular, the interaction between the C2B-domain and the plasma membrane has been shown to determine the speed of calcium-induced SV fusion (Evans et al., 2015). Knock-out of synaptotagmin leads to the loss of calcium evoked synchronous release but at the same time to an increase in spontaneous SV fusion (Geppert et al., 1994). Thus, although the P_r_ of the synapse is decreased in terms of AP-induced synaptic transmission, the P_r_ of single SVs in synaptotagmin knock-out synapses is actually increased, as they fuse spontaneously. The loss of synchronous release and the concomitant increase in spontaneous fusion can be attributed to discrete parts of the C2-domain. Expression of synaptotagmin 2 lacking the entire C2B-domain in the calyx of Held of synaptotagmin 2 knock-out mice has no effect on synaptic release. However, a synaptotagmin 2 version that contains a C2B-domain that is unable to bind calcium, decreases the rate of spontaneous release although synchronous release is still impaired. This partial rescue is presumably mediated by the basic amino acid residues of the C2B-domain that bind to the assembled SNARE complex (Kochubey and Schneggenburger, 2011). However, ablation of basic amino acid residues of the C2B-domain that interact with the plasma membrane has no effect on the spontaneous release rate, but reduces synchronous release by impairing the positional priming of SVs (Young and Neher, 2009). Interestingly, the fusion of SVs seems also to depend on the composition of the plasma membrane at the site of fusion. Both synaptotagmin and syntaxin 1 interact with the plasma membrane lipid phosphatidylinositol 4,5-bisphosphate that clusters syntaxin 1 and increases the calcium sensitivity of the synaptotagmin C2B-domain (van den Bogaart et al., 2011, 2012; Honigmann et al., 2013; Rizzoli, 2014). Thus, the principal calcium sensor synaptotagmin is a key player in determining the P_r_ of a SV, as it integrates multiple steps of the release process and thereby sets the P_r_ according to the current microenvironmental conditions of the SV.

Another component of the AZ that regulates P_r_ is the G-protein-coupled receptor kinase-interacting protein 1 (GIT1) that has been implicated in the recycling of SVs (Podufall et al., 2014). However, knock-out of GIT1 at the calyx of Held results in an increase in P_r_ through a yet unknown mechanism that does not involve the size of the RRP or the calcium influx into the terminal. Interestingly, the knock-out of the closely related GIT2 had no effect on synaptic transmission, and simultaneous knock-out of both GIT isoforms phenocopied the GIT1 knock-out, arguing against an involvement of GIT2 in regulation of P_r_ (Montesinos et al., 2015).

Thus, the complicated molecular machinery of the SNARE complex and its associated proteins play a crucial role in regulating the P_r_ of the synapse in multiple ways: (1) the assembly of SNARE complex determines the number of SVs available for fusion; (2) binding of additional proteins (e.g., Munc13) regulates the energy barrier of SV fusion; (3) SNARE complex components are involved in positional priming of SVs; and (4) binding of synaptotagmin to calcium, the plasma membrane and the SNARE complex regulates SV fusion.

## SV Proteins Regulating P_r_

In addition to the SNARE complex protein synaptobrevin and the SV proteins involved in docking and priming of SVs (e.g., Rab3, see above), a number of SV proteins have been implicated in regulating P_r_, with the vesicular glutamate transporter VgluT representing the most prominent example. VGluT exists in three isoforms (VGluT1–3) with distinct expression patterns in the mammalian brain (Edwards, 2007). Synapses expressing VGluT1 show a relatively low P_r_, whereas synapses expressing VGluT2 have a high P_r_ (Fremeau et al., 2004). This regulation of P_r_ is directly mediated by the transporter itself, as domain swapping between VGluT1 and VGluT2 can transpose the P_r_ setting properties. The low P_r_ of VGluT1 carrying SVs may be due the ability of VGluT1 but not VGluT2 to bind to endophilin A1 and inhibit an endophilin-mediated enhancement of SV fusion (Weston et al., 2011). Furthermore, it has been shown that the filling state of neurotransmitter in the SV, which correlates with the number of transporter proteins on the SV, regulates P_r_ (Herman et al., 2014). The filling state of the SV in turn is not only regulated by the neurotransmitter transporters, but also involves the establishment of a proton gradient across the SV membrane by the v-ATPase (Edwards, 2007) and the activity of a K^+^/H^+^-exchanger to make this electrochemical gradient useable for the neurotransmitter loading (Goh et al., 2011).

In addition to VGluTs, Mover, a recently discovered vertebrate-specific SV protein (Kremer et al., 2007; Ahmed et al., 2013), has been implicated in the regulation of P_r_ (Körber et al., 2015). Mover binds to the AZ scaffolding protein bassoon and the acute knock-down of Mover at the calyx of Held leads to an increase in P_r_ by increasing the calcium sensitivity of release. Interestingly, the knock-down of CaM, to which Mover can bind, has also been shown to affect P_r_, although the knock-down of CaM rather yields a decrease in P_r_ (Pang et al., 2010).

Thus, components of the molecular machinery of the SV are not only involved in docking, priming and the generation of the fusion machine, but also regulate vesicular P_r_ by setting the filling state of the SV or changing the calcium-dependence of release.

## Asynchronous Release of Neurotransmitter

**Figure 1:**
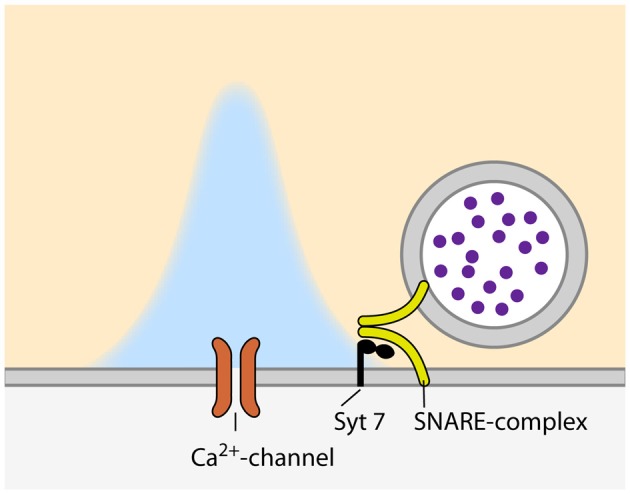
**Configuration of synaptic vesicles (SVs) and voltage-gated calcium channels (VGCCs) during asynchronous release.** Please note the larger distance between the VGCC and calcium sensor as compared to synchronous relase (Figure 2).

The release of neurotransmitter from docked and primed SVs can occur in different modes; spontaneous, synchronous and asynchronous. The P_r_ of a synapse is normally defined as the probability of a SV to be released synchronously, immediately after the arrival of an AP at the presynapse. Nevertheless, some synapses show prominent asynchronous, AP-triggered but delayed, neurotransmitter release (for a recent review, see Kaeser and Regehr, 2014). Thus, there is a probability that a given SV will not be released immediately after the arrival of the AP, but asynchronously, with a potentially different P_r_. The specific mechanisms and molecular machines involved in this form of release are poorly understood. VAMP4 has been proposed to substitute for synaptobrevin in the SNARE complex of SVs that are destined to asynchronous release (Raingo et al., 2012) and synapsin II has been suggested to be involved in asynchronous SV release at GABAergic synapses (Medrihan et al., 2013). Furthermore, there is accumulating evidence that synchronous and asynchronous release are triggered by different calcium sensors. Whereas synchronous release depends on synaptotagmin 1, 2 or 9 (Xu et al., 2007), the identity of the calcium sensor for asynchronous release is still under debate. Two proteins have been proposed to trigger asynchronous release: synaptotagmin 7 and Doc2 (Yao et al., 2011; Bacaj et al., 2013, respectively). Doc2 has been originally shown to be involved in spontaneous neurotransmitter release (Groffen et al., 2010; Pang et al., 2011). However, a recent controversial study proposed Doc2 as a sensor for asynchronous release, as Doc2 knock-down in hippocampal cultured neurons resulted in a shift towards synchronous release upon intense stimulation (Yao et al., 2011). Synaptotagmin 1 knock-out synapses on the other hand, show prominent asynchronous release that can be completely abolished by simultaneous knock-down of synaptotagmin 7, suggesting that synaptotagmin 7 acts as the calcium sensor for asynchronous release (Bacaj et al., 2013). Also, synaptotagmin 7 has been proposed recently to act as a calcium sensor for SV replenishment in synapses of cultured hippocampal neurons (Liu C. et al., 2014), a process known to be at least partially calcium-dependent (e.g., Wang and Kaczmarek, 1998). However, this effect could not be confirmed at synapses between rod bipolar and AII amacrine cells in the retina, where synaptotagmin 7 again acts as a sensor for asynchronous release, but has no effect on SV replenishment (Luo et al., 2015). Of note, asynchronous release does not only rely on a specialized calcium sensor, but also on additional components of the AZ. A recent study at the calyx of Held showed that the knock-out of complexin 1 results in a profound increase in asynchronous release which goes along with a reduction in both synchronous and spontaneous release (Chang et al., 2015). Moreover, the protein adapter complex AP-3, which is thought to be involved in the regeneration of SVs from synaptic endosomes (Voglmaier et al., 2006), has been shown to contribute to asynchronous release at hippocampal mossy fiber synapses (Evstratova et al., 2014). Taken together, asynchronous release constitutes another, as of now poorly understood, layer of P_r_ regulation that depends on several molecular machines of the presynaptic terminal. Nevertheless, it seems to rely on a specialized fusion machinery, in particular a separate calcium sensor, most likely synaptotagmin 7 (Figure 1).

## The Regulation of P_r_ by Calcium

**Figure 2:**
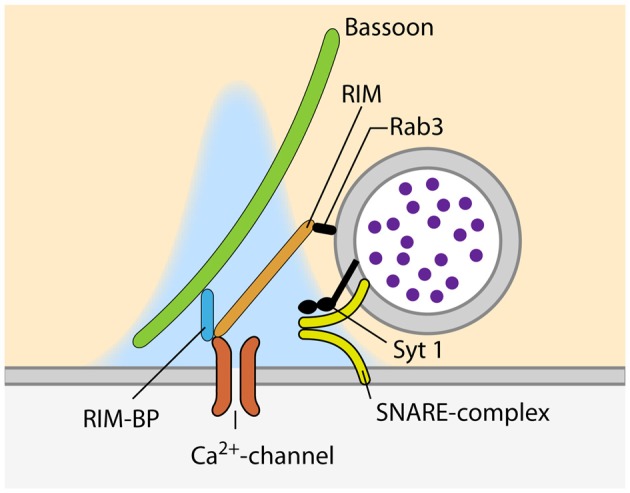
**Proteins involved in the recruitment of VGCC and SVs to sites of SV fusion during synchronous release**.

In addition to the availability of primed SVs at the site of release, the regulation of calcium influx into the presynaptic compartment is key to define vesicular P_r_. Calcium influx occurs via VGCCs precisely located within the AZs. The VGCCs are organized in clusters and their number per cluster correlates with the P_r_ of the release site (Holderith et al., 2012; Sheng et al., 2012; Nakamura et al., 2015; but see Scimemi and Diamond, 2012). The arrival of an AP opens the VGCCs and leads to the influx of calcium ions into the presynaptic terminal, where they very briefly build-up a transient domain of elevated calcium concentration high enough to trigger synaptotagmin-dependent release of SVs (Figure 2). The effective calcium concentration at the SV-bound calcium sensor directly controls the P_r_ of a given SV, as it decides whether this SV is released in response to the arrival of an AP and the opening the VGCCs. Endogenous buffers, passive diffusion and the active extrusion mechanisms lead to a rapid break down of the calcium domain, so that the fusion of SVs is confined to the vicinity of the VGCC cluster (e.g., Borst and Sakmann, 1996; Eggermann et al., 2012; Neher and Taschenberger, 2013; Babai et al., 2014). How the SVs are arranged with respect to the VGCC cluster is still a matter of debate and may vary according to the synapse type and developmental stage. In fast-spiking GABAergic interneurons of the hippocampus, SVs are thought be placed 10–20 nm away from the VGCCs (Bucurenciu et al., 2008; Arai and Jonas, 2014), whereas the coupling distance is ~75 nm at hippocampal mossy fiber to CA3 synapses (Vyleta and Jonas, 2014). At the calyx of Held, varying values have been estimated for mature synapses: 5–25 nm (Chen et al., 2015), 15–30 nm (Nakamura et al., 2015) and >30 nm (Keller et al., 2015). This coupling distance is subject to developmental regulation and has been shown to decrease at the calyx of Held (Fedchyshyn and Wang, 2005; Kochubey et al., 2009; Leão and von Gersdorff, 2009; Nakamura et al., 2015) and the parallel fiber to Purkinje cell synapse in the cerebellum (Baur et al., 2015), possibly by a septin 5-dependent mechanism (Yang et al., 2010). Different coupling distances can also lead to differences in short-term plasticity (STP) during ongoing synaptic activity due to differences in calcium buffering (e.g., Eggermann and Jonas, 2012; Pan and Ryan, 2012; Vyleta and Jonas, 2014). Hence, calcium channels need to be placed in a specific arrangement within a narrow distance range relative to the SVs to ensure precise setting of P_r_.

## Recruitment of Calcium Channels to the AZ

At the site of SV fusion, the AZ cytomatrix represents a molecular machine that consists mostly of large scaffolding proteins, ensuring that SVs and VGCCs are located at the optimal distance for timed SV fusion (Südhof, 2012, 2013). One of the key molecules involved in the coupling between SVs and calcium channels is RIM, as it binds to SVs via Rab3, and directly to calcium channels of the P/Q-(Ca_v_2.1) and N-type (Ca_v_2.2) but not L-type (Ca_v_1.1–3; Figure 2; Wang et al., 1997; Kaeser et al., 2011, respectively). Simultaneous genetic deletion of RIM1 and 2 leads to a profound loss of VGCCs at the AZ of cultured hippocampal and the calyx of Held, which greatly decreases the P_r_ of the synapses (Kaeser et al., 2011; Han et al., 2011, respectively). The importance of different RIM isoforms seems to vary between synapses. RIM1 and 2 have been shown to be functionally redundant at the calyx of Held as the deletion of either of the isoforms had no effect on VGCC recruitment to the AZ (Han et al., 2015). However, at cerebellar granule cell terminals, loss of the RIM1α isoform leads to reduced calcium influx due to reduced channel abundance (Kintscher et al., 2013). Interestingly, RIM abundance at the AZ is regulated via its recruitment by liprin-α (Spangler et al., 2013) as well as by its SUMOylation, which is required for VGCC recruitment but dispensable for SV binding (Girach et al., 2013). Furthermore, RIM1 is regulated in an activity dependent manner. Prolonged silencing of neuronal activity in hippocampal cultured neurons, strongly increasing P_r_ (Murthy et al., 2001), results in an upregulation of RIM (Lazarevic et al., 2011). This observation supports the idea that the amount of RIM present at the synapse sets its P_r_, probably by determining the number of VGCCs at the AZ. Besides RIM, the RIM-binding protein (RIM-BP) that binds to RIM as well as to VGCCs, has been proposed to be involved in VGCC clustering at the AZ (Wang et al., 2000; Hibino et al., 2002; Figure 2). Moreover, RIM-BPs bind to the presynaptic scaffolding protein bassoon and a loss of this interaction has been shown to lead to a change in VGCC composition, with P/Q-type channels being lost from the AZ and replaced by N-type channels. The presence of RIM-BPs is thus necessary for the specific recruitment of P/Q-type channels to the SV release sites (Davydova et al., 2014). The simultaneous loss of both isoforms of RIM-BP (RIM-BP1 and 2) results in a reduction in EPSC amplitude, both in hippocampal cultures and the calyx of Held, but does not change the kinetic properties and amplitude of presynaptic calcium currents. Instead, the loss of RIM-BPs causes an increased variability of SV fusion in response to an AP, due to defects in the coupling of VGCCs and SVs (Acuna et al., 2015). In addition to the scaffolding proteins of the cytomatrix, a recent study reported an increase in coupling distance at the calyx of Held of complexin 1 knock-out mice (Chang et al., 2015). Interestingly, the abundance of VGCCs at the AZ is not only controlled by interactions with components of the cytomatrix of the AZ, but also by the VGCC α2δ-subunit. The expression level of this subunit directly sets the number of P/Q-type channels at the presynaptic compartment, thereby determining the number of SVs fusing upon AP-arrival and therefore, defining P_r_ (Hoppa et al., 2012).

## Direct Modulation of Calcium influx Through VGCCs

The calcium influx into the presynaptic compartment through VGCCs is one of the key parameters determining P_r_ (Ermolyuk et al., 2012) and therefore not only regulated by the means of channel abundance, but also by direct modulation of the calcium flux through the channel. The presynaptic scaffolding proteins ELKS1 and 2 (Südhof, 2012) are two of the proteins that directly affect calcium flux without affecting VGCC abundance. Simultaneous genetic ablation of both ELKS isoforms in hippocampal neurons results in a strongly decreased P_r_ at inhibitory terminals which is due to a decrease in AP-induced calcium influx (Liu H. et al., 2014). Another presynaptic protein that regulates the functional properties of VGCCs is Munc13. Simultaneous knock-down of Munc13-1 and 2 in hippocampal cultured neurons decreases the amount of AP-induced calcium influx. This is mediated by the C2B-domain of Munc13, which regulates the kinetic properties, such as inactivation and refractory period, of the VGCCs and therefore has a strong influence on the P_r_ during ongoing synaptic activity (Calloway et al., 2015). Thus, proteins of the presynaptic cytomatrix of the AZ are not only recruiting VGCCs to the AZ, but are also involved in regulating their functional properties and thereby the P_r_ of the synapse.

Additionally, calcium influx through VGCCs is regulated in a calcium-dependent manner: depending on the recent history of synaptic activity, calcium influx can either be facilitated or inhibited. This VGCC modulation is directly dependent on the interaction of VGCCs, at least of the P/Q-type, with CaM and other calcium binding proteins of the presynaptic compartment (reviewed by Catterall and Few, 2008; Ben-Johny and Yue, 2014). Calcium-dependent CaM binding to the VGCCs C-terminus initially facilitates calcium influx and then promotes channel inactivation, thereby inhibiting calcium influx during prolonged depolarization (Lee et al., 1999, 2000; DeMaria et al., 2001). However, CaM has recently been shown to also bind to the P/Q-type VGCC in the non-activated, calcium-free form, causing robust facilitation of the calcium current. This is released by subsequent calcium binding to CaM, resulting in an apparent VGCC inhibition (Adams et al., 2014). Furthermore, other presynaptic calcium-binding protein have been shown to interact with VGCCs and to modulate their gating properties, and in turn, calcium influx and P_r_. CaBP-1, NCS-1 and VLIP-2 all modulate calcium-dependent facilitation and inactivation of the P/Q-type VGCC, albeit at different extents (Lee et al., 2002; Tsujimoto et al., 2002; Lautermilch et al., 2005, respectively). This calcium/CaM-dependent modulation of calcium influx through VGCCs has profound impact on STP and thus information transfer at a variety of synapses (Catterall et al., 2013).

Apart from proteins of the AZ, VGCCs are modulated by a variety of extracellular signals. Activation of presynaptic GABA_B_Rs for example results in a profound decrease in calcium influx at various synpses due to direct interactions of activated G-proteins with the VGCCs (Dittman and Regehr, 1996; Kajikawa et al., 2001; Sakaba and Neher, 2003a; Wang et al., 2013; Kupferschmidt and Lovinger, 2015). Inhibition of VGCCs by direct interaction with G-proteins upon activation of G-protein-coupled receptors (GPCRs) provides a general mechanism to regulate presynaptic calcium influx during ongoing synaptic activity. The activation of presynaptic A1 adenosine receptors (e.g., Dittman and Regehr, 1996; Wong et al., 2006), α2-noradrenergic receptors (e.g., Leão and Von Gersdorff, 2002) cannabinoid receptors (e.g., Kushmerick et al., 2004) and metabotropic glutamate receptors (e.g., von Gersdorff et al., 1997; Renden et al., 2005; Kupferschmidt and Lovinger, 2015) results in VGCC inhibition, and thereby reduced calcium influx and P_r_, too. Furthermore, VGCCs are also regulated by the neurotrophin BDNF (brain-derived neurotrophic factor), which inhibits the activation of VGCCs at the calyx of Held (Baydyuk et al., 2015). Of note, BDNF has also been proposed to regulate the developmental switch in VGCC type at Purkinje cell output synapses (Miki et al., 2013).

Interestingly, VGCCs can additionally be modulated by direct modification. At least N-type VGCCs are regulated by balanced phosphorylation/dephosphorylation mediated by CDK5 and CaN (Su et al., 2012; Kim and Ryan, 2013). And moreover, the α2δ subunit of the VGCC has been shown to set-up VGCC for triggering SV fusion by binding extracellular metal ions (Hoppa et al., 2012). Thus, the calcium flux through VGCC is controlled by intracellular factors, like AZ proteins, as well as by extracellular signals such as neuromodulators that act via presynaptically expressed GPCRs.

## Indirect Modulation of Calcium influx Through VGCCs

Calcium influx through VGCCs, and therefore P_r_, is not only regulated by direct modulation of VGCC function as described above, but is also dependent on the resting membrane potential (RMP) of the presynaptic plasma membrane, which has a large effect on the activation of the VGCCs. The RMP of the presynaptic terminal is determined by the expression of specialized ion channels that collaborate to set the optimal RMP according to the current functional needs of the synapse. However, as direct measurements of ion channel function are only possible at few synapses in the mammalian CNS, most of our knowledge on this topic arises from the calyx of Held, although it may also apply to other synapses. At the calyx of Held, the RMP is set and regulated by the potassium channel subunit K_v_7.5 (Huang and Trussell, 2011), the hyperpolarization-activated cyclic nucleotide-gated (HCN) channel (Cuttle et al., 2001; Kim et al., 2007), the Na^+^/K^+^-ATPase (Kim et al., 2007) and a persistent sodium channel of currently unknown origin (Huang and Trussell, 2008). Shifting the RMP to more depolarized potentials by either blocking K_v_7.5 or activating HCN channels results in the facilitation of VGCC opening and thereby to increases in calcium influx and SV release upon arrival of an AP. A hyperpolarizing shift in the RMP can occur after prolonged high-frequency firing at the calyx of Held, due to the activation of the Na^+^/K^+^-ATPase containing the α3 subunit. Interestingly, this hyperpolarization is counteracted by the activation of HCN channels (Kim et al., 2007). Along the same lines, the RMP of the calyx of Held can be regulated by the activation of presynaptic glycine receptors (GlyRs). Since the chloride concentration in the calyx of Held is relatively high, activation of GlyRs depolarizes the presynaptic terminal and therefore facilitates VGCC opening and increases and P_r_ (Turecek and Trussell, 2001; Price and Trussell, 2006). Regulation of the presynaptic RMP hence sets the P_r_ of the synapse by regulating the efficacy of calcium influx into the presynaptic compartment.

Moreover, the calcium influx into the presynaptic terminal and the P_r_ can be regulated by the modification of the AP-width: at the calyx of Held, the AP-width narrows during synaptic maturation, concomitant with a decrease in P_r_ (Taschenberger et al., 2002). This mechanism depends on the modulation of presynaptic voltage-gated potassium channels (K_v_s). At hippocampal cultured synapses, the modulation of AP-width can counteract changes in presynaptic VGCC abundance to keep P_r_ in these synapses constant. This mechanism depends on K_v_3.1 and K_v_1 (Hoppa et al., 2014), both of which have also been shown to be present at the calyx of Held terminal (Ishikawa et al., 2003). K_v_s are regulated by ongoing synaptic activity which allows them to adjust the P_r_ according to the current status of the circuit. K_v_s have been shown to undergo activity-dependent facilitation during high frequency firing at the calyx of Held, in order to constrain the duration of the presynaptic AP and thus calcium influx and SV release (Yang et al., 2014). However, the mechanisms by which these regulations of K_v_-function occur are largely unknown. A recent study has shown that the membrane-derived lipid arachidonic acid can act as a retrograde messenger to broaden the AP by modulation of K_v_-function at the hippocampal mossy fiber to CA3 synapse (Carta et al., 2014). Taken together, in addition to direct regulation of VGCC abundance or function, indirect effects originating from either the RMP or the AP-width also constitute alternative, powerful ways of regulating P_r_.

## Regulation of P_r_ During Short-term Plasticity

The P_r_ is not only regulated by the molecular machines involved in the recruitment, docking and priming of SVs as well as the positioning and regulation of VGCCs, but also by changes in the presynaptic environment during ongoing activity. Stretches of high synaptic activity induce STP in many mammalian CNS synapses. STP can occur, depending on the synapse and the rate of activity, either as short-term facilitation (STF) or short-term depression (STD). STP subsides within seconds and is an important factor in information processing, as e.g., synapses undergoing STF are more likely to transmit information later in a sequence of presynaptic activity, thereby acting as a high-pass filter, whereas synapses showing STD have a higher initial P_r_ and act as a low-pass filter by having a higher chance to pass-on the information at the beginning of a sequence of synaptic events. STF is generally thought to depend on the build-up of residual calcium in the presynaptic terminal resulting in a spread of the transient calcium domain and an increase in calcium concentration sufficiently high to trigger neurtransmitter release at SVs that would normally not reach the calcium concentration threshold for fusion. Additionally, calcium-dependent modulation of VGCCs has profound effects on STF, as facilitaion of calcium influx can contibute to the build-up of such residual calcium levels (Catterall and Few, 2008; Catterall et al., 2013). A recent study reported STF at synapses between Purkinje cells that relies primarily on calcium dependent fascilitation of calcium influx (Díaz-Rojas et al., 2015). However, different mechanisms of STF have been shown to operate at certain synapses, e.g., at the cerebellar parallel fiber to Purkinje cell synapse, additional release sites are recruited during STF (Brachtendorf et al., 2015). Moreover, a recent study reported STF to be absent from synaptotagmin 7-KO mice, suggesting that synaptotagmin 7 acts as a calcium sensor specific for STF (Jackman et al., 2016). STD on the other hand has been suggested to be caused by a depletion of the RRP that cannot be balanced by the replenishment machinery (for review, see Zucker and Regehr, 2002).

The mechanisms by which changes in P_r_ regulate STP are diverse. In essence, every step interfering with the molecular machines described so far can cause alterations in STP. Therefore, a large variety of presynaptic proteins have been implicated in the regulation of STP and the P_r_ of both, a SV to fuse and the synapse as a whole to transmit information. For example: (1) alterations in the rate of SNARE complex formation, correlated to the availability of syntaxin 1, have a profound effect on STD in hippocampal cultured neurons (Arancillo et al., 2013); (2) the slowing of recruitment of SVs to the AZ in the absence of synapsins also accelerates STD (Vasileva et al., 2012).

The availability of release-ready SVs in general is one of the main factors determining STP and thus synaptic P_r_ during ongoing synaptic activity. Therefore, impairing of SV endocytosis leads in general to severe effects on STP, mostly to strong STD. Effective endocytosis and thereby reformation of SVs depends on proteins of a variety of different molecular machines. It involves e.g., SV proteins as synaptophysin and α-synuclein (Kwon and Chapman, 2011; Vargas et al., 2014, respectively), but also proteins of the endocytic core machinery such as stonin 2, clathrin, AP180 or dynamin (Hua et al., 2013; López-Murcia et al., 2014; Kaempf et al., 2015; Koo et al., 2015, respectively). The detailed mechanisms by which SV endocytosis is coupled to exocytosis and STP have recently been reviewed elsewhere (Haucke et al., 2011).

Recently, the view that replenishment of SVs to the site of release is the limiting factor during STD, has been challenged. Studies at the calyx of Held revealed that the block of endocytosis has an immediate effect on SV fusion that is too fast to be explained by the impairment of SV regeneration (Hosoi et al., 2009). Therefore, it has been proposed that the rate-limiting step in SV release under ongoing activity is the clearance of the release site of proteins deposited there by the previously fusing SV (Hosoi et al., 2009). This release site clearance model assumes a fixed number of release sites of which most, but not all, are occupied by a primed SV at the beginning of a sequence of activity. Upon the arrival of APs, more and more of the primed SVs fuse with the plasma membrane and clog the release sites with their protein content. In order to refill the release sites with newly primed SVs, this “debris” first needs to be removed to clear the release site. This clearance then is the rate-limiting step during ongoing SV release and hence has a strong impact of the regulation of P_r_ (Neher, 2010).

## Post-Tetanic Potentiation

A special case of STP is post-tetanic potentiation (PTP) that can be observed at many synapses in the CNS. PTP is induced by prolonged intense stimulation, typically 100 Hz for 5–10 s, and lasts for tens of seconds to minutes (Zucker and Regehr, 2002). It combines an increase in RRP size and in P_r_ (Habets and Borst, 2005; Korogod et al., 2005; Fioravante et al., 2011). Moreover, an increase in quantal size (the postsynaptic current induced by the release of neurotransmitter from a single SV) during PTP has been reported (He et al., 2009; Xue and Wu, 2010; Fioravante et al., 2011), although other groups did not detect this effect (Habets and Borst, 2005; Korogod et al., 2005). The increase in RRP size and P_r_, but not in quantal size, depend on the activation of the calcium-dependent protein kinase C (PKC) subunits PKCα, PKCβ and PKCγ (Korogod et al., 2007; Xue and Wu, 2010; Fioravante et al., 2011; Chu et al., 2014) of which PKCβ acts as a calcium sensor (Fioravante et al., 2014). Additionally, the mechanisms by which PKC mediates PTP have been reported to be developmentally regulated. PKCα and PKCβ are responsible for ~80% of PTP at the more mature calyx of Held (P11–14) primarily by an increase in RRP size (Fioravante et al., 2011). At the juvenile calyx of Held (P8–10) however, PKCγ and PKCβ mediate PTP by increasing the P_r_ of individual SVs (Chu et al., 2014). Nevertheless, PKC is not the only protein contributing to PTP as calyces of Held lacking PKCα and PKCβ still express 20% of PTP (Fioravante et al., 2011). These alternative mechanisms of PTP may involve MLCK, which has also been shown to be involved in the induction of PTP at the calyx of Held (Lee et al., 2008). Of note, PKC can also be activated by the continuous activation of presynaptic GlyRs, likewise increasing RRP size and P_r_ (Chu et al., 2012). The mechanism by which PKC mediates PTP has recently been shown to involve the phosphorylation of Munc18-1, which then increases the vesicular P_r_ in a yet unknown manner (Genc et al., 2014). Once the tetanic stimulus is over and the intracellular calcium concentration returns to baseline, PKC is deactivated and the phosphorylation of Munc18-1 is removed by protein phosphatases 1 and 2A, thereby terminating PTP (Genc et al., 2014).

## Conclusions

**Figure 3:**
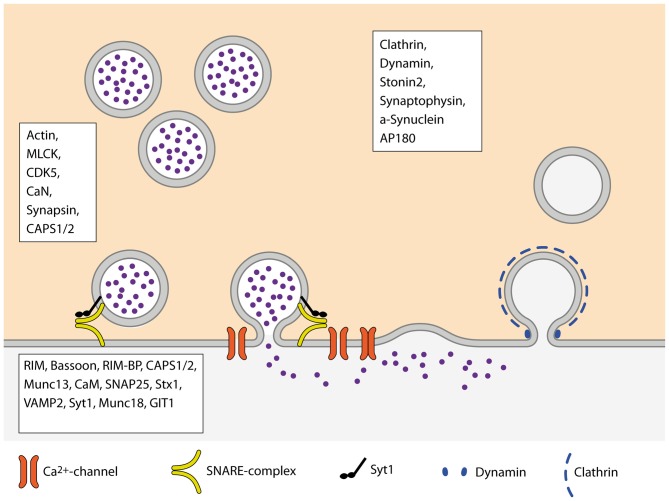
**Schematic overview of the SV cycle.** Boxes name proteins of the respective steps of the cycle that are involved in the regulation of P_r_.

The P_r_ of SVs to be released upon the arrival of an AP, constituting the successful transfer of information across the synapse, is one of the crucial parameters underlying brain function. However, the mechanisms and molecular machines involved in its regulation are still not fully understood. One of the problems in understanding P_r_ arises from the large variety of processes that impact on it, as reviewed here (Figure 3). At some point, most of the presynaptic machinery will affect P_r_ in one way or the other, may it be the availability of release-ready SVs, regulation of VGCCs or some other mechanism. An additional layer of complexity in understanding P_r_ derives from the fact that P_r_ is highly variable between synapses, different types of synapses as well as synapses originating from the same axon (Atwood and Karunanithi, 2002; Branco and Staras, 2009). Further studies will therefore focus on elucidating the regulatory mechanisms that set the P_r_ of SVs at a given synapse according to the current demands of the circuit.

## Author Contributions

CK and TK wrote the article.

## Conflict of Interest Statement

The authors declare that the research was conducted in the absence of any commercial or financial relationships that could be construed as a potential conflict of interest.
